# Predicting hematoma expansion in acute spontaneous intracerebral hemorrhage: integrating clinical factors with a multitask deep learning model for non-contrast head CT

**DOI:** 10.1007/s00234-024-03298-y

**Published:** 2024-02-10

**Authors:** Hyochul Lee, Junhyeok Lee, Joon Jang, Inpyeong Hwang, Kyu Sung Choi, Jung Hyun Park, Jin Wook Chung, Seung Hong Choi

**Affiliations:** 1 Interdisciplinary Program in Cancer Biology, Seoul National University College of Medicine, Seoul, 03080 Republic of Korea; 2 Department of Radiology, Seoul National University Hospital, 101 Daehak-Ro, Jongno-Gu, Seoul, 03080 Republic of Korea; 3 Department of Biomedical Sciences, Seoul National University, Seoul, 03080 Korea; 4 Department of Radiology, Seoul National University College of Medicine, 101 Daehak-Ro, Jongno-Gu, Seoul, 03080 Republic of Korea; 5 Artificial Intelligence Collaborative Network, Department of Radiology, Seoul National University Hospital, Seoul, 03080 Republic of Korea; 6 Department of Radiology, Seoul Metropolitan Government Seoul National University Boramae Medical Center, Seoul, 07061 South Korea; 7 Center for Nanoparticle Research, Institute for Basic Science (IBS), Seoul, 08826 Republic of Korea

**Keywords:** Acute intracerebral hemorrhage, Deep learning, Hematoma expansion, Computed tomography, Clinical finding

## Abstract

**Purpose:**

To predict hematoma growth in intracerebral hemorrhage patients by combining clinical findings with non-contrast CT imaging features analyzed through deep learning.

**Methods:**

Three models were developed to predict hematoma expansion (HE) in 572 patients. We utilized multi-task learning for both hematoma segmentation and prediction of expansion: the Image-to-HE model processed hematoma slices, extracting features and computing a normalized DL score for HE prediction. The Clinical-to-HE model utilized multivariate logistic regression on clinical variables. The Integrated-to-HE model combined image-derived and clinical data. Significant clinical variables were selected using forward selection in logistic regression. The two models incorporating clinical variables were statistically validated.

**Results:**

For hematoma detection, the diagnostic performance of the developed multi-task model was excellent (AUC, 0.99). For expansion prediction, three models were evaluated for predicting HE. The Image-to-HE model achieved an accuracy of 67.3%, sensitivity of 81.0%, specificity of 64.0%, and an AUC of 0.76. The Clinical-to-HE model registered an accuracy of 74.8%, sensitivity of 81.0%, specificity of 73.3%, and an AUC of 0.81. The Integrated-to-HE model, merging both image and clinical data, excelled with an accuracy of 81.3%, sensitivity of 76.2%, specificity of 82.6%, and an AUC of 0.83. The Integrated-to-HE model, aligning closest to the diagonal line and indicating the highest level of calibration, showcases superior performance in predicting HE outcomes among the three models.

**Conclusion:**

The integration of clinical findings with non-contrast CT imaging features analyzed through deep learning showed the potential for improving the prediction of HE in acute spontaneous intracerebral hemorrhage patients.

**Supplementary Information:**

The online version contains supplementary material available at 10.1007/s00234-024-03298-y.

## Introduction

Spontaneous intracerebral hemorrhage (ICH) accounts for 10–15% of stroke [1] with a high mortality rate, specifically 40% at 1 month of onset [2], necessitating urgent and accurate diagnosis and treatment decision. According to recent reviews, hematoma expansion, occurring in 20% of patients with ICH, is a significant contributor to early neurological impairment and unfavorable prognosis following intracerebral hemorrhage [3, 4].

Previous research has identified several factors influencing hematoma expansion. These include imaging features, notably specific CT markers such as the spot sign observed in CT angiography and the black hole sign in non-contrast CT, alongside the initial size of the hematoma [5]. Additionally, clinical features such as laboratory parameters (e.g., fibrinogen levels and platelet counts), anticoagulation status, time to hospital admission, and alcohol consumption have been recognized as contributing factors [1].

While deep learning has shown remarkable performance in various tasks related to hematoma, such as detection, segmentation, quantification, and classification of subdural hematoma (SDH), epidural hematoma (EDH), and ICH [6–9], its application in predicting hematoma expansion has been limited. Previous studies have utilized clinical variables and Support vector machines (SVM) for prediction of hematoma expansion [1]. To our knowledge, although there have been qualitative assessments of the diagnostic value of CT angiography (CTA) spot signs and CT black hole signs, there have been only one study employing deep learning for predicting hematoma expansion using CT images [10], which have focused solely on image-based predictions, without incorporating or correlating with clinical variables. However, there has been few existing studies have combined imaging features with other clinical variables to predict hematoma expansion [10–12].

This study aimed to explore whether deep learning can extract significant clinical indicators from non-contrast CT and to assess the relative impact of these extracted factors when compared to known clinical indicators, employing a large-scale dataset to develop and validate a deep learning model for the prediction of hematoma expansion, thereby contributing to more effective and personalized treatment strategies.

## Methods and materials

### Study population

**Fig. 1:**
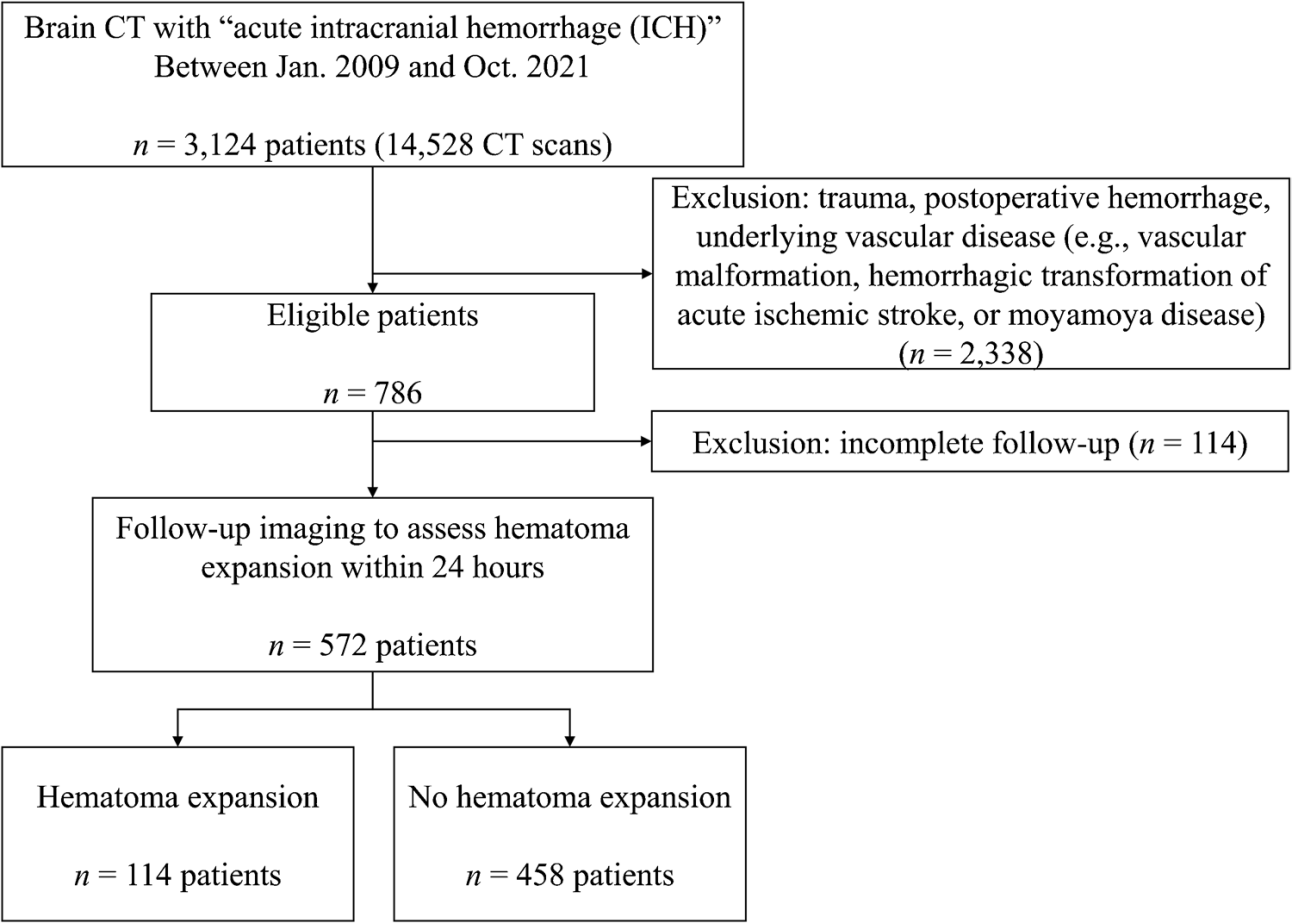
Flowchart indicating the included and excluded patients

This single-center, retrospective study was approved by the institutional review board, and the informed consent was waived (IRB No. 2111–165-1276). We enrolled patients who performed non-contrast head CT scans between January 2009 and October 2021 in Seoul National University Hospital. We included patients who were 19 years or older, initially diagnosed with spontaneous acute intracerebral hemorrhage by non-contrast head CT, and subsequent follow-up images within 24 h to evaluate hematoma size change. The exclusion criteria were (1) patients with traumatic brain hemorrhage, (2) patients with secondary ICH (e.g., vascular malformation, hemorrhagic transformation of acute ischemic stroke, or moyamoya disease), (3) lack of follow-up images within 24 h or underwent surgical treatment before subsequent follow-up images, or (4) suboptimal CT image quality. Finally, 572 patients were enrolled for a hematoma expansion dataset. The detailed patient enrollment process was described in Fig. 1.

### Head CT acquisition and image interpretation

The non-contrast head CT scans were performed with one of the CT scanners of various vendors, which varied over time within our institution: 320-slice multidetector CT scanner (Aquilion ONE, Canon Medical Systems), 256-slice scanner (iCT256, Philips Medical Systems), 64-slice scanner (Brilliance 64, Philips Medical Systems; SOMATOM Definition, Siemens Healthineers; Discovery 750 HD, GE Healthcare), or 16-slice scanner (Sensation 16, Siemens Healthineers). The image acquisition parameters were relatively constant as follows: peak tube voltage, 120 kV; tube current, 150 ~ 400 mAs with automatic exposure control; reconstruction matrix size, 512 × 512; reconstruction field of view, 24 cm × 24 cm and slice thickness, 3 mm or 5 mm. All CT images were reconstructed using a kernel appropriate for brain evaluation (Supplementary Table 1).

One radiologist with 12 years of experience in neuroradiology (I.H.) measured hematoma volume in initial and follow-up CT images using picture archiving and communication system workstation (INFINITT M6 and Xelis, INFINITT Healthcare, Seoul, Korea). If the patient underwent more than two follow-up imaging, the hematoma volume was measured based on the images that appeared largest upon visual inspection. On multi-planar reformatted images, the investigators measured largest diameter (D1) and two perpendicular diameters (D_2_ and D_3_) using electric caliper. The volume of the hematoma was determined using the ellipsoid approximation as follows: volume = 0.523 × D_1_ × D_2_ × D_3_. Hematoma expansion was defined as ≥ 6 mL absolute or ≥ 33% relative increase on follow-up [13]. The same investigator (I.H.) also semi-automatically determined hematoma region-of-interest mask on the initial CT scans for training the segmentation model, using threshold method in NordicICE software (version 4.1.2, NordicNeuroLab, Bergen, Norway).

### Clinical variables

The collected clinical variables included sex, age, Glasgow coma scale (GCS) group, systolic blood pressure (SBP), diastolic blood pressure (DBP), fibrinogen, international normalized ratio (INR), platelet count (platelet), antiplatelet use, time to initial CT scan, activated partial thromboplastin time (aPTT), initial hematoma volume, percent hematoma change, and absolute hematoma change. GCS scores were categorized based on their summation: severe brain injury (≤ 8), mild injury (9–12), and minor injury (≥ 13). Time to initial CT scan was defined as the duration between symptom manifestation and the CT scan.

### Image preprocessing

To accommodate hematoma characteristics in non-contrast CT, all CT images were normalized within a range of 0 to 100 Hounsfield units. The images were resized to a standard dimension of 512 × 512. Data augmentation was conducted using the “ShiftScaleRotate” and “RandomScale” functionalities from the Python “albumentation” library [14].

### Dataset

We prepared a large dataset for developing multitask deep learning model for (1) hematoma segmentation and (2) prediction of hematoma expansion. For the hematoma slice classification and segmentation model, details are provided in the supplemental materials. For the HE prediction model, the trained hematoma slice classification model was used to extract slices indicative of hematoma. From a total of 24,238 slices from 572 patients, 6044 slices were identified from 569 patients as having hematoma. The training set comprised 4834 slices from 458 patients, while the test set consisted of 1210 slices from 111 patients. We summarized the distribution and statistical analysis of HE and non-HE patients scanned with each type of CT scanner in the datasets in Supplementary Table 2. Stratification was applied based on the distribution of HE and non-HE cases. In the training set of 4834 images, 1152 slices came from 92 HE patients, and 3682 slices were from 366 non-HE patients. In the test set, from the total of 1219 images, 261 were from 22 HE patients and 949 from 89 non-HE patients. To compare the results of HE prediction including and excluding clinical variables, we considered unavailable clinical elements and excluded them. Consequently, the final data set for evaluation of models employed 107 patients: 21 HE patients and 86 non-HE patients (Supplementary Table 3).

### Deep learning algorithm

**Fig. 2:**
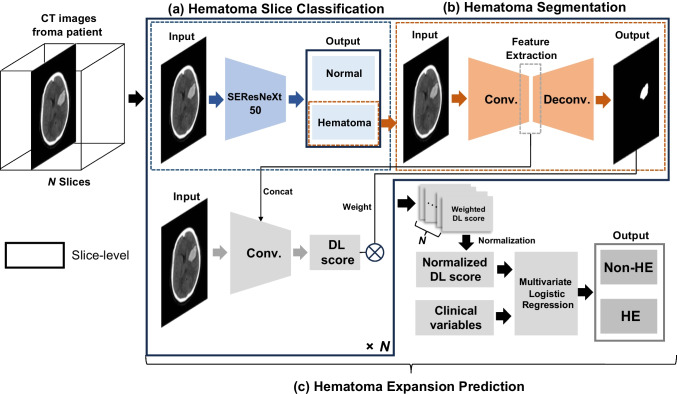
The workflow of hematoma expansion prediction model. This figure illustrates a multi-task deep learning approach for hematoma expansion prediction in non-contrast head CT images, encompassing slice classification, segmentation, and expansion prediction of hematoma. **a** The classification phase utilizes the SEResNeXt-50 model, enhanced with attention mechanisms to distinguish normal from hematoma-containing slices. **b** The segmentation phase using the U-Net model facilitates feature extraction associated with hematomas and utilizes the size of the segmented hematoma regions as a weighting factor in subsequent prediction models. **c** For prediction of HE, the convolutional neural network employs CT image and feature extracted from the segmentation model. The DL score through the model is calculated with weighting factor derived from the size of the hematoma region. The process for acquiring the normalized DL score is performed on a per-slice basis for each patient. Consequently, multivariate logistic regression is utilized, combining the normalized DL score with clinical variables obtained from the patient, to predict the HE. Note: CT = computed tomography, HE = hematoma expansion, DL = deep learning

For hematoma slice classification model, we designed a model to distinguish hematoma slices from a patient’s non-contrast head CT images (Fig. 2a). A pre-trained “SEResNeXt-50” architecture, enriched with attention mechanisms attention [15–17], was employed. SEResNeXt-50, an enhanced variant of ResNeXt-50 which itself is based on VGG and ResNets architectures, combines repetitive layering with a split-transform-merge strategy for deep feature learning in image classification, and integrates Squeeze-and-Excitation blocks to emphasize crucial features in convolutional layers, significantly improving image classification performance [15, 16]. The model was implemented using PyTorch, with a learning rate of 1e-4, a weight decay of 1e-6, and a batch size of 8. This fine-tuned model accepts 2D CT images of size 512 × 512 as input and determines the presence or absence of a hematoma.

For hematoma segmentation model development, we targeted the segmentation of hematoma regions within images containing hematoma (Fig. 2b). The prevalent U-Net architecture for segmentation was adopted [18]. This model operates with a learning rate of 0.001 and utilizes a combined loss function derived from the Dice coefficient and binary cross entropy.

For expansion prediction model, we aimed to predict hematoma expansion (HE) by training our model to process hematoma images as inputs and predict the HE as outputs (Fig. 2c). The model, termed the Image-to-HE model, relies exclusively on hematoma images for HE prediction. It accepts a single slice as input and extracts features post-encoder from the trained hematoma segmentation model. After feature extraction, a concatenation process in the convolutional neural network deduces the HE probability, referred to as the DL score. The model then uses the hematoma region size, produced by the trained hematoma segmentation model, as a weight. This weight is introduced to reflect the hematoma size across multiple CT images for a single patient, ensuring proportionality to the hematoma’s size. The Image-to-HE model predicts a patient-specific HE outcome using the normalized DL score (nDL score).

The Clinical-to-HE model forecasts HE using multivariate logistic regression on clinical variables, notably without incorporating image data.

The Integrated Image and Clinical-to-HE model (Integrated-to-HE) incorporates both image-derived and clinical variables. This comprehensive model uses the normalized DL score as a regressor within a multivariate logistic regression framework, aligning it with clinical variables to predict patient-specific HE.

### Statistical analysis

Only clinically significant variables, as determined by a univariable logistic regression *p* value, were used as inputs for the models. For the selection of clinical variables, a logistic regression analysis with a forward selection method was employed. The variables found to be statistically significant were chosen for the model. These statistical analyses were conducted using statistical software (MedCalc, Mariakerke, Belgium). The performance of the classification and prediction models was evaluated using accuracy, sensitivity, specificity, and area under the curve (AUC) metrics. The segmentation model’s performance was assessed using the Dice coefficient metric. Model performances were compared using accuracy, sensitivity, specificity, and AUC scores on the test set. Also, model performances were assessed using AUC to measure the ability to distinguish between classes through the receiver operating characteristic (ROC) curve and calibration plots to compare predicted probabilities against actual outcomes. Models were compared using their log-likelihoods, with odds ratios and 95% confidence intervals calculated for each variable; the overall significance between models was determined through the likelihood ratio test (LRT), which follows a chi-squared distribution. The model fit and complexity of the Clinical-to-HE and Integrated-to-HE models were assessed using Akaike’s information criterion (AIC) with lower values indicating a better fit.

### Qualitative analysis

For analysis of our deep learning model, we applied the gradient-weighted class activation mapping (Grad-CAM) technique as a method to understand and visualize which image regions our deep learning model focuses on for predicting hematoma expansion [19]. This technique creates a localization map by utilizing the gradients flowing into the final convolution layer of the model, highlighting the important regions for analysis. We implemented Grad-CAM from the last convolution layer of the Integrated-to-HE model to visually represent the area deemed significant by the model in non-contrast head CT images for hematoma expansion prediction.

## Results

### Clinical characteristics and univariable analysis of clinical variable

The clinical characteristics and results of the univariable logistic regression for each clinical variable are provided in Table 1. By univariable analysis for prediction of HE, age (OR, 0.98; *p* < 0.001), moderate and severe GCS group (OR, 2.52 and 4.24, respectively, all *p* < 0.001), fibrinogen (OR, 1.00; *p* = 0.034), INR (OR, 1.44; *p* = 0.026), platelet (OR, 1.00; *p* = 0.037), and initial hematoma volume (OR, 1.02; *p* < 0.001) were the significant predictors. Sex, blood pressures, antiplatelet use, aPTT, and time to the initial CT scan were not found to be significant (all *p* > 0.05).

**Table 1:** Cohort characteristics and statistical results of univariable logistic regression. Data are presented mean ± standard deviation, percentage

Clinical variable		Cohort characteristic		Univariable analysis	
		Non-HE (*n* = 455)	HE (*n* = 114)	OR [95% CI]	*P* value
Sex (male:female)		262: 193	75: 39	1.42 [0.92, 2.18]	0.112
Age (years)		63 ± 14	58 ± 14	0.98 [0.96, 0.99]	< 0.001*
GCS group	Mild	310	53	1.00	
	Moderate	65	28	2.52 [1.48, 4.28]	< 0.001*
	Severe	40	29	4.24 [2.42, 7.42]	< 0.001*
SBP (mmHg)		166 ± 37	162 ± 40	1.00 [0.99, 1.00]	0.285
DBP (mmHg)		92 ± 21	91 ± 20	1.00 [0.99, 1.01]	0.547
Fibrinogen (mg/dL)		330 ± 107	305 ± 129	1.00 [1.00, 1.00]	0.034*
INR		1.09 ± 0.60	1.27 ± 0.66	1.44 [1.04, 2.00]	0.026*
Platelet (× 10^9^/L)		211 ± 75	192 ± 106	1.00 [1.00, 1.00]	0.037*
Antiplatelet use		95 (21%)	27 (24%)	1.08 [0.66, 1.78]	0.756
Time to initial CT scan (h)		6.2 ± 6.7	5.7 ± 6.5	1.00 [1.00, 1.00]	0.518
aPTT (s)		31.1 ± 6.1	35.4 ± 35.7	1.02 [0.99, 1.06]	0.141
Initial hematoma volume (mm^3^)		18.1 ± 22.7	37.0 ± 34.6	1.02 [1.01, 1.04]	< 0.001*
Percent hematoma change (%)		6.2 ± 26.2	325.1 ± 687.5		
Absolute hematoma change (mm^3^)		0.3 ± 4.9	28.4 ± 22.7		

*HE* Hematoma expansion, *OR* odds ratio, *CI* confidence interval, *GCS* Glasgow coma scale, *SBP* systolic blood pressure, *INR* international normalized ratio, *aPTT* activated partial thromboplastin time, *P < 0.05. Missing values (non-HE, HE); GCS group = 525/569 (415/455, 110/114), SBP = 525/569 (420/455, 105/114), DBP = 524/569 (422/455, 112/114), fibrinogen = 534/569 (422/455, 112/114), INR = 536/569 (424/455, 112/114), platelet = 532/569 (424/455, 112/114), antiplatelet use = 473/569 (420/455, 112/114), time to initial CT scan = 537/569 (429/455, 108/114), aPTT = 522/569 (412/455, 110/114).

### Multivariable analysis of models

In the multivariable logistic regression analysis aimed at determining the selection of clinical variables, the forward selection method was employed. Among the clinical variables including age, GCS group, fibrinogen, INR, and platelet, only age, GCS group, and fibrinogen demonstrated statistical significance and were thus selected for the model. Since the DL score includes information about hematoma size as an image feature, initial hematoma volume was excluded as a clinical variable for the models.

In the model development phase, we assessed the significance of clinical variables by evaluating their odds ratios and associated *p* values across different models. Table 2 provides a detailed comparison of the odds ratios and corresponding *p* values for each clinical variable within three distinct models: Image-to-HE, Clinical-to-HE, and Integrated-to-HE. For age, the odds ratio across all three models remained consistent at 0.98. However, the significance of this ratio varied between models with *p* values of 0.021 and 0.044 for the Clinical-to-HE and Integrated-to-HE models, respectively, suggesting a mild yet significant effect of age on the outcome. In contrast, the Clinical-to-HE model did not indicate a significant impact of age.

**Table 2:** Statistical results of clinical variables and normalized deep learning score across Image-to-HE, Clinical-to-HE, and Integrated-to-HE models

Variable		Model					
		Image-to-HE		Clinical-to-HE		Integrated-to-HE	
		OR [95% CI]	*P* value	OR [95% CI]	*P* value	OR [95% CI]	*P* value
Age				0.98 [0.96, 1.00]	0.021*	0.98 [0.96, 1.00]	0.044*
GCS group	Mild			1.00		1.00	
	Moderate			2.85 [1.50, 5.42]	0.001*	2.11 [1.06, 4.20]	0.033*
	Severe			4.30 [2.26, 8.17]	< 0.001*	1.92 [0.92, 3.98]	0.081
Fibrinogen				1.00 [1.00, 1.00]	0.147	1.00 [1.00, 1.00]	0.114
nDL score		1.12 [1.08, 1.15]	< 0.001*			1.10 [1.07, 1.14]	< 0.001*

*HE* Hematoma expansion, *OR* odds ratio, *CI* confidence interval, *GCS* Glasgow coma scale, *nDL score* normalized deep learning score, *P < 0.05.

In the GCS group category, the “Moderate” condition exhibited an odds ratio of 2.85 in the Clinical-to-HE model and 2.11 in the Integrated-to-HE model, both with significant *p* values of 0.001 and 0.033, respectively. The “Severe” condition presented an odds ratio of 4.30 in the Clinical-to-HE model with a *p* value less than 0.001, indicating a strong correlation. However, in the Integrated-to-HE model, the association was not statistically significant. For fibrinogen, the odds ratio remained at 1.00 across all models, showing no particular trend or significance. The nDL score in the Image-to-HE model was 1.12, with a highly significant *p* value of less than 0.001. Similarly, in the Integrated-to-HE model, the odds ratio was 1.10 with a *p* value less than 0.001, suggesting a robust correlation between the nDL score and the outcome.

### Performances in multitask deep learning model: hematoma detection and segmentation

For the task of hematoma slice classification, the model put forth an excellent performance with an accuracy of 97.7%. Further reinforcing its diagnostic prowess, the model displayed a sensitivity of 93.6% and a specificity of 99.5%. Notably, the AUC of the receiver operating characteristic curve for this model stood high at 99.4%. For the task of hematoma segmentation, our model displayed a Dice similarity coefficient of 77.2%, indicating a reasonably good overlap between the predicted segmentation and the ground truth.

### Performances in multitask deep learning model: prediction of hematoma expansion

**Fig. 3:**
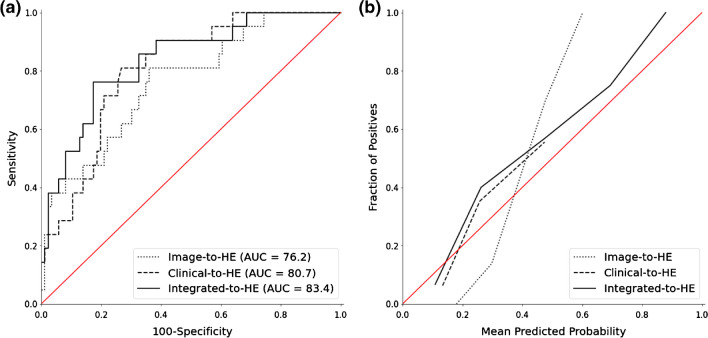
**a** ROC curves for Image-to-HE, Clinical-to-HE, and Integrated-to-HE models, showing the respective AUC values. **b** Calibration plots comparing the predicted probabilities to actual outcomes for the three models. Note: ROC = receiver operating characteristic, HE = hematoma expansion, AUC = area under the curve

Table 3 summarizes the models’ diagnostic performance on the test set. The Image-to-HE model displayed an AUC of 0.76 (95% CI: 0.64, 0.89). Using optimal thresholds, the model detected conditions with a sensitivity of 81.0% (17 of 21 cases). The overall accuracy stood at 67.3% (72 of 107 cases), and specificity was noted at 64.0% (55 of 86 cases). Additionally, the confidence intervals for accuracy and sensitivity ranged from 57.9 to 75.5% and 60.0 to 92.3%, respectively. In comparison, the Clinical-to-HE model achieved an AUC of 0.81 (95% CI: 0.69, 0.93). With the best thresholds in place, it showed a sensitivity of 81.0% (17 of 21 cases) and a specificity of 73.3% (63 of 86 cases). The total accuracy of predictions was 74.8% (80 of 107 cases). The confidence intervals for accuracy and sensitivity were between 65.8 to 82.0% and 60.0 to 92.3%, respectively. Lastly, the Integrated-to-HE model, combining clinical and image data, showcased an AUC of 0.83 (95% CI: 0.72, 0.95). It detected conditions with a sensitivity of 76.2% (16 of 21 cases). The model’s overall accuracy was the highest at 81.3% (87 of 107 cases), and its specificity reached 82.6% (71 of 86 cases). Confidence intervals for accuracy and sensitivity were found to be 72.9 to 87.6% and 54.9 to 89.4%, respectively. The AUC serves as a measure of the model's ability to distinguish between the classes (Fig. 3a). The Image-to-HE model has an AUC of 0.76, which indicates a good level of separability, but it puts behind the other two models. The Clinical-to-HE model, with an AUC of 0.81, offers a better performance than the Image-to-HE model. The Integrated-to-HE model stands out with an AUC of 0.83, implying that the integration of both image and clinical data provides superior classification capabilities. The calibration plot contrasts the predicted probabilities to the actual outcomes for the three models (Fig. 3b). A perfectly calibrated model would have its curve lying on the diagonal red line. The Image-to-HE model deviates notably from the ideal, suggesting some discrepancies between the predicted probabilities and actual outcomes. The Clinical-to-HE model shows closer adherence to the diagonal, denoting improved calibration. The Integrated-to-HE model appears closest to the diagonal line, indicating the highest level of calibration among the three.

**Table 3:** Comparisons of model performances on the test set

Model	Accuracy [95% CI]	Sensitivity [95% CI]	Specificity [95% CI]	AUC [95% CI]
Image-to-HE	67.3 (72/107) [57.9, 75.5]	81.0 (17/21) [60.0, 92.3]	64.0 (55/86) [63.5, 88.7]	0.76 [0.64, 0.89]
Clinical-to-HE	74.8 (80/107) [65.8, 82.0]	81.0 (17/21) [60.0, 92.3]	73.3 (63/86) [63.1, 81.5]	0.81 [0.69, 0.93]
Integrated-to-HE	81.3 (87/107) [72.9, 87.6]	76.2 (16/21) [54.9, 89.4]	82.6 (71/86) [73.2, 89.1]	0.83 [0.72, 0.95]

*HE* Hematoma expansion, *CI* confidence interval, *AUC* area under the curve.

### Comparative statistical analysis of Clinical-to-HE and Integrated-to-HE models

The *P* value derived from the LRT. This difference follows a chi-squared distribution, facilitating tests to determine if the two models exhibit statistically significant variations. Both models showed significant differences, with a *P* value less than the threshold (*P* < 0.001). The AIC is employed to assess model fit and complexity. The Clinical-to-HE model had an AIC of 400.72, while the Integrated-to-HE model registered an AIC of 369.93. A lower AIC value signifies a better fit. Thus, the observed AIC values suggest that the Integrated-to-HE model offers a superior fit to the data, potentially due to fewer parameters or a more streamlined model complexity, compared to the Clinical-to-HE model.

### Qualitative results of Integrated-to-HE model with Grad-CAM

**Fig. 4:**
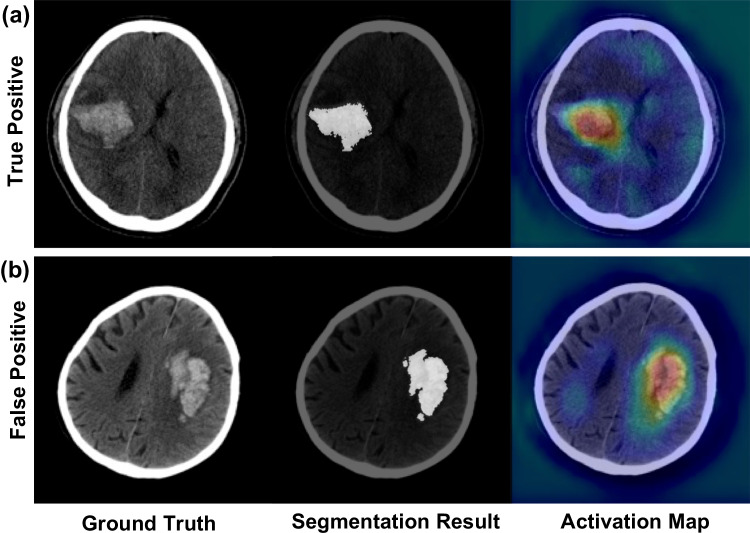
Visualization of hematoma segmentation and activation mapping. Representative axial CT images of two patients with intracerebral hemorrhages. **a** True positive case (top row), and **b** false positive case (bottom row). The left column showcases the original CT scans, highlighting the hematoma. The middle column displays the segmented region of the hematoma, emphasized in white. The right column illustrates the activation maps

In the evaluation of the Integrated-to-HE model on the test set, we analyzed both true positive and false positive cases, as illustrated in Fig. 4. One observation from a patient with confirmed hematoma expansion was the model’s proficiency in hematoma segmentation (Fig. 4a, middle). Leveraging feature extraction, and utilizing Grad-CAM, we discerned pronounced activation within the hematoma region (Fig. 4a, right). The model successfully predicted hematoma expansion with the derived nDL score and supplemented clinical variables.

## Discussion

In this study, we devised a multitask deep learning model that integrates clinical variables with non-contrast CT images to forecast hematoma expansion in patients with spontaneous ICH. Notably, our model demonstrated superior performance when compared to a model that solely relied on clinical variables. These findings underscore the potential benefits of leveraging multitask deep learning approaches to enhance predictive accuracy for hematoma expansion.

The incidence of hematoma expansion in this study was observed at 20% (21 out of 107 patients), which is marginally below the 26 to 36% range reported in other studies [20–22]. This difference can be attributed to exclusion of patients from our study who receives follow-up imaging due to the early surgical interventions necessitated by neurological deterioration in hematoma patients.

Previous research has suggested associations between various clinical variables and hematoma expansion [3]. However, through univariable analysis, it was demonstrated that age [23], GCS group, fibrinogen [24], INR, and platelet [25, 26] are meaningful variables [27]. In our study, age, GCS group, and fibrinogen were shown to be significant by multivariable logistic regression, as presented in Table 1. Yet, the significance of these variables varied across models. Specifically, the Integrated-to-HE model indicated age, GCS group, and nDL score as significant predictors, as seen in Table 2.

Although age and fibrinogen did not show substantial influence based on their OR, it is essential to consider that the significance might be influenced by the limited patient dataset used for the study, which might not account for meaningful differences among patient groups in terms of hematoma expansion.

The GCS score has long been recognized as a crucial indicator [24]. Patients with initial bleeding tend to have poorer functional outcomes and an increased risk of expansion. Notably, the association of the “Moderate” GCS group with the outcome is statistically significant, with an OR of 2.11. The *p* value of 0.081 for the “Severe” GCS group in our study might indicate the limited ability to detect significant results due to the small number of patients in this group. Additionally, the inclusion of the DL score in our analysis suggests it may capture some of the variations linked to the severe GCS, which could affect the overall results of the model.

The Integrated-to-HE model highlights the predictive value of the nDL score. A noteworthy observation is that with each unit increase in the nDL score, there is a 10% surge in the odds of the outcome occurring, a result that is both prominent and statistically significant. This suggests that the outcomes derived from multitask deep learning effectively serve as meaningful variables, reflecting the association with hematoma expansion predictions.

The performance comparison distinctly underscores the superior capabilities of the Integrated-to-HE model over the Clinical-to-HE model (Table 3). In previous studies, using clinical data and non-contrast CT for HE prediction, the AUC was 0.79, and the accuracy was 77.5% [12]. In another study, the AUC was 0.80 [11]. In another study, an AUC of 0.78 was demonstrated [10]. Using multivariable logistic regression, the results showed an AUC of 0.72 for the development cohort and 0.77 for the independent validation cohort [28]. In our study, the Integrated-to-HE model achieved a remarkable AUC of 0.83, presenting better results when compared to previous research. Moreover, statistical comparisons also reveal significant differences between the Clinical-to-HE and Integrated-to-HE models. The calibration plot and AIC metrics further cement the fact that the Integrated-to-HE model offers a better fit to the data (Fig. 3). While models utilizing solely imaging data demonstrate limitations in predicting hematoma expansion, it becomes evident that leveraging both clinical variables and imaging data enhances prediction accuracy. This is evinced by the markedly improved results of the Integrated-to-HE model, suggesting the paramount importance of a combined data approach in clinical prognostication.

In qualitative evaluation of the Integrated-to-HE model on the test set, as depicted in Fig. 4, we observed that the model, proficient in hematoma segmentation associated with feature extraction, demonstrated distinct activation within the hematoma region [19]. This was further elucidated using Grad-CAM, which allowed us to identify activation in areas significant for hematoma expansion prediction. Consequently, the model successfully predicted hematoma expansion using derived nDL scores and supplemented clinical variables. The application of Grad-CAM provides visual explanations from deep networks associated with insights from the model for hematoma expansion prediction. This approach aids in addressing the challenges in understanding the decision-making process of the model [19].

Conversely, in a case where the algorithm misclassified a non-expanding hematoma as expanding, the CT image manifested potential indicators, such as the CT swirl sign (Fig. 4b, left). This could elucidate the model’s predilection for an expansion prediction. Importantly, the algorithm exhibited superior segmentation capabilities, accurately demarcating the hematoma region (Fig. 4b, middle). Concurrently, the activation was predominantly localized within the segmented areas, underscoring the model’s specificity (Fig. 4b, right). Identifying the CT spot sign from images is crucial for predicting hematoma expansion [29, 30]. However, relying solely on images for prediction introduces complexities, making it challenging. Even radiologists can be deceived by these images, as they may exhibit features indicative of expansion, potentially leading to false positives.

For robust interpretability of multitask deep learning model, we employed a consistency loss function between predicted segmentation and label of hematoma expansion, which ensures that reducing false positive prediction of hematoma expansion (Fig. 2) [31]. This enhancement demonstrates the model’s capability to extract relevant features from CT images, suggesting its potential use in hematoma expansion prediction.

In the field of hematoma research, deep learning has been predominantly used for tasks like detection and classification. Although many laboratory tests, especially in emergency room settings, can be time-consuming, CT scans are consistently conducted and readily available. Our study presents a unique methodological approach. We enhanced performance by integrating imaging features with clinical variables using logistic regression, particularly utilizing data that does not rely on variables from laboratory tests. Additionally, we incorporated multitask learning into our image-only model, facilitating the extraction of more robust features. The two approaches underscore the distinctive contribution of our study in predicting hematoma expansion.

Our study, conducted within a single center, ensured consistency but limited the diversity of patient data, raising questions about the model’s performance in varied contexts without external validation. The dataset used had limitations, and a more comprehensive dataset could enhance our model’s training and predictive capabilities. Our model was designed to exclude normal patients, increasing specificity for hematoma expansion prediction but potentially challenging its broader applicability. Although our primary aim was to improve hematoma expansion prediction using multitask learning, future work could benefit from including a normal patient cohort to enhance the model’s adaptability and depth. Furthermore, we aimed to include CT scans from all major scanner vendors with variable scanner channels installed in our institution to reduce the risk of overfitting in our single-center study design.

In conclusion, the integration of clinical findings with non-contrast CT imaging features analyzed through deep learning showed the potential for improving the prediction of HE in acute spontaneous ICH patients. Although our integrated model did not reach statistical significance in terms of diagnostic performance, it demonstrated a statistically better fit in multivariable logistic regression analysis compared to the clinical-only model. Hence, our approach may offer synergistic benefits over using only conventional clinical variables, highlighting the potential of utilizing readily available information such as the GCS and CT images. This integrated strategy underscores a promising direction for personalized treatment strategies and improved patient outcomes in ICH management.

### Supplementary Information

Below is the link to the electronic supplementary material.

Supplementary file1 (PDF 157 KB)

## Data Availability

The training, validation, and test sets for this study are protected patient information. The data supporting the findings of this study are available within the paper and its supplementary materials. The raw data of the study and code base for deep-learning framework in the study are available from the corresponding author, upon reasonable request.
