# Supplementary material for: Predicting hematoma expansion in acute spontaneous intracerebral hemorrhage: integrating clinical factors with a multitask deep learning model for non-contrast head CT
Source: Neuroradiology. 2024 Feb 10;66(4):577–87. doi: 10.1007/s00234-024-03298-y (PMC10937749; doi:10.1007/s00234-024-03298-y)
Supplement: Supplementary file 1 — Supplementary file1 (PDF 157 KB) [file 234_2024_3298_MOESM1_ESM.pdf]

Supplementary Materials

Dataset for hematoma slice classification and segmentation

For the training of the classification model discerning hematoma slices, the datasets were stratified based on the number of hematoma slices per patient. Subset of 336 patients was used for this task, resulting in a training set comprising 10,827 slices from 294 patients and a test set with 1,507 slices from 42 patients. In the training set, 3,461 slices were identified as hematoma, while 7,366 slices were categorized as non-hematoma. For the test set, 472 and 1,035 slices were labeled as hematoma and non-hematoma, respectively. Both training and test sets maintained a consistent distribution between hematoma and non-hematoma class ratios.

For hematoma segmentation, data from 334 of 336 patients previously used in hematoma slice classification were employed. The training phase utilized only slices with associated masks. From the dataset, 3,933 slices were sourced; 3,231 images from patients formed the training set, with the remaining 702 images from 52 patients designated as the test set.

Supplementary Table

Supplementary Table 1. CT Scanners and convolution kernels

CT Scanner	Image Reconstruction Convolution Kernel*
Aquilion ONE (Canon)	FC68
Brilliance 64 (Philips)	UB
SOMATOM Definition (Siemens)	H30 or H31
Sensation 16 (Siemens)	H30
iCT 256 (Philips)	UB
Discovery 750 HD (GE)	STANDARD

*Vendor provided convolution kernel name

Supplementary Table 2. HE and non-HE patient distribution and statistical analysis by CT scanner type in datasets with significant differences.

CT Scanner	Train (n=461)		Test (n=111)	
	Non-HE (n=369)	HE (n=92)	Non-HE (n=89)	HE (n=22)
Aquilion ONE (Canon)	140	37	43	7
Brilliance 64 (Philips)	93	27	17	8
SOMATOM Definition (Siemens)	42	6	5	1
Sensation 16 (Siemens)	33	4	11	2
iCT 256 (Philips)	29	9	9	2
Discovery 750 HD (GE)	32	9	4	2

The Chi-squared statistic is approximately 16.3. The P-value obtained from Pearson's Chi-squared test is 0.362. Note: HE = hematoma expansion.

Supplementary Table 3. Distribution of hematoma and non-hematoma expansion cases in training and test sets excluding unavailable data.

Group	Train	Test	Total
HE Patients (Slices)	92 (1,152)	22 (261)	114 (1,413)
Non-HE Patients (Slices)	366 (3,682)	89 (949)	455 (1,210)
Total Patients (Slices)	458 (4,834)	111 (1,210)	569 (6,044)

Note: HE = hematoma expansion
